# Supplementary material for: Transcriptome Analysis Reveals Vimentin-Induced Disruption of Cell–Cell Associations Augments Breast Cancer Cell Migration
Source: Cells. 2022 Dec 13;11(24):4035. doi: 10.3390/cells11244035 (PMC9776984; doi:10.3390/cells11244035)
Supplement: Supplementary file 1 [file cells-11-04035-s001.zip › DEGs and enrichment analysis.zip!/DEGs and enrichment analysis/WT_VIMvsVector KEGG/ALL/WT_VIMvsVector_ALL.pathway_map.zip!/WT_VIMvsVector_ALL.pathway_map/src/hsa04068.html]

hsa04068


- hsa:9133

- Up regulated genes

ENSG00000157456(1.60409075038332)

- hsa:5347

- Up regulated genes

ENSG00000166851(2.38554234415561)

- hsa:23710

- Down regulated genes

ENSG00000139112(-1.85791559298273)

- hsa:7042

- Down regulated genes

ENSG00000092969(-2.32734226266555)

Close
